# Modelling Neuroinflammation In Vitro: A Tool to Test the Potential Neuroprotective Effect of Anti-Inflammatory Agents

**DOI:** 10.1371/journal.pone.0045227

**Published:** 2012-09-20

**Authors:** Núria Gresa-Arribas, Cristina Viéitez, Guido Dentesano, Joan Serratosa, Josep Saura, Carme Solà

**Affiliations:** 1 Department of Cerebral Ischemia and Neurodegeneration, Institut d’Investigacions Biomèdiques de Barcelona-Consejo Superior de Investigaciones Científicas (CSIC), Institut d’Investigacions Biomèdiques August-Pi i Sunyer (IDIBAPS), Barcelona, Spain; 2 Biochemistry and Molecular Biology Unit, School of Medicine, University of Barcelona, IDIBAPS, Barcelona, Spain; Emory University, United States of America

## Abstract

Neuron-microglia co-cultures treated with pro-inflammatory agents are a useful tool to study neuroinflammation *in vitro*, where to test the potential neuroprotective effect of anti-inflammatory compounds. However, a great diversity of experimental conditions can be found in the literature, making difficult to select the working conditions when considering this approach for the first time. We compared the use of neuron-primary microglia and neuron-BV2 cells (a microglial cell line) co-cultures, using different neuron:microglia ratios, treatments and time post-treatment to induce glial activation and derived neurotoxicity. We show that each model requires different experimental conditions, but that both neuron-BV2 and neuron-primary microglia LPS/IFN-γ-treated co-cultures are good to study the potential neuroprotective effect of anti-inflammatory agents. The contribution of different pro-inflammatory parameters in the neurotoxicity induced by reactive microglial cells was determined. IL-10 pre-treatment completely inhibited LPS/IFN-γ-induced TNF-α and IL-6 release, and COX-2 expression both in BV2 and primary microglial cultures, but not NO production and iNOS expression. However, LPS/IFN-γ induced neurotoxicity was not inhibited in IL-10 pre-treated co-cultures. The inhibition of NO production using the specific iNOS inhibitor 1400 W totally abolished the neurotoxic effect of LPS/IFN-γ, suggesting a major role for NO in the neurotoxic effect of activated microglia. Consequently, among the anti-inflammatory agents, special attention should be paid to compounds that inhibit NO production.

## Introduction

The presence of reactive glia or neuroinflammation has been described in all neurodegenerative diseases, and glial activation, especially microglial activation, may contribute to the neuropathology observed [1], [2]. The inhibition of neuroinflammation has been postulated as a putative target in the treatment of neurodegenerative diseases, and research has focused on the study of potential neuroprotective effects of anti-inflammatory agents in experimental models of neurodegeneration occurring in the presence of reactive glia.

In spite of the obvious differences between microglia *in vivo* and *in vitro*, cultured microglia constitute a powerful tool to study how different agents can modify microglial activation in response to known CNS active agents [3]. *In vitro* approaches are often used to characterize molecules and pathways involved in microglial activation. Numerous studies have considered different types of glial cultures treated with lipopolysaccharide (LPS) or pro-inflammatory cytokines to characterize glial activation and to test the potential anti-inflammatory effect of candidate compounds. Experimental *in vitro* models of neurotoxicity induced by reactive glia are useful in turn to test the potential neuroprotective effect of anti-inflammatory agents, which can then be assayed *in vivo* [4]–[8]. The microglial content in rodent primary mixed neuron-glia cultures is usually low, due to the fact that brain embryos (embryonic day 15–16) are generally used to obtain neurons in culture while glial cultures are optimally obtained from 2–3 days postnatal brains. Although microglial cells populate the rodent brain around embryonic day 10, a marked increase in the number of microglia is observed in the CNS in the first few days after birth [9]. As microglial cells are believed to play a critical role in the neurotoxicity induced by reactive glia [1], [2], the low amount of microglia present makes difficult to study glial activation and the resulting neurotoxicity in primary mixed neuron-glia cultures. An exception is primary mesencephalic cultures, which show a higher amount of microglial cells than cultures obtained from other brain regions due to the fact that the substantia nigra is the brain area with the highest microglia content [10], [11]. However, mesencephalic cultures are mostly used when the interest is focused on dopaminergic neurons. An experimental strategy to overcome the low amount of microglial cells usually detected in primary mixed neuron-glia cultures is the use of co-culture approaches, where variable amounts of microglial cells are added on top of mixed neuron-glia cultures. Experimental conditions using co-cultures show great variability between laboratories [12]–[16]. The neuron:microglia ratio, the decision to use either primary cultures or different cell lines, the animal source of the glial cultures (usually mouse or rat) and the stimulus inducing glial activation all determine differences in the degree of neurotoxicity induced by reactive glial cells in neuron-glia co-cultures.

In the present study, we establish two experimental models of neuroinflammation *in vitro*, comparing the use of primary microglial cells and the microglial cell line BV2. Neuronal death is produced in mouse neuron-microglia co-cultures after the induction of glial activation using LPS/interferon (IFN)-γ, but different experimental conditions are needed when BV2 or primary microglia are considered. We studied the contribution of different pro-inflammatory parameters in the neurotoxicity induced by reactive microglia in the co-cultures. To this end we pre-treated neuron-microglia co-cultures with the classical anti-inflammatory cytokine interleukin (IL)-10 and the inducible nitric oxide synthase (iNOS) inhibitor 1400 W. We point out nitric oxide (NO) as one of the main targets to be considered in anti-inflammatory strategies leading to neuroprotection.

## Methods

### Ethics Statement

Experiments were carried out in accordance with the Guidelines of the European Union Council (86/609/EU) and following the Spanish regulations (BOE 67/8509-12, 1988) for the use of laboratory animals, and the protocols were approved by the Ethics Committee from the Barcelona University and the Ethics and Scientific Committees from the CSIC (Permit Numbers: 217/11 and 218/11).

**Figure 1:**
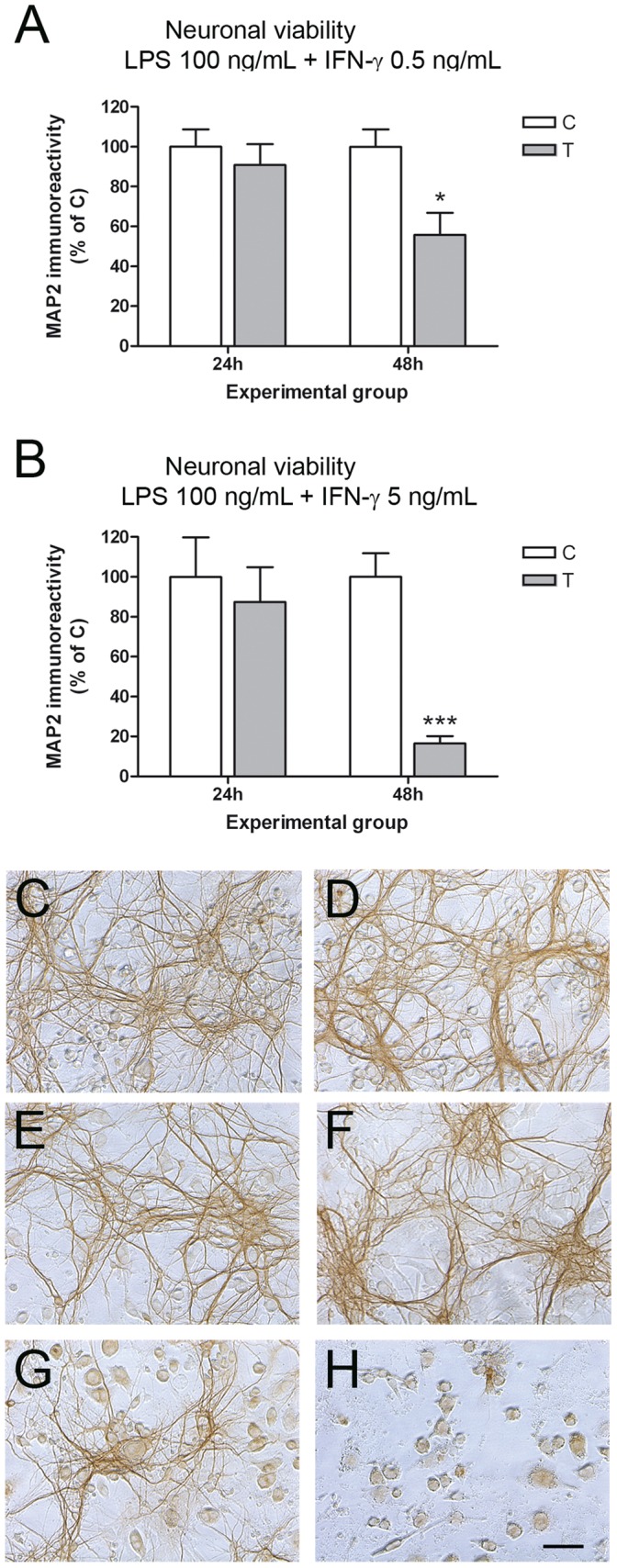
Glial activation and resulting neurotoxicity in neuron-BV2 co-cultures. Neuronal viability (MAP2-ABTS-ELISA assay) in neuron-BV2 co-cultures 24 h or 48 h after treatment with 100 ng/mL LPS +0.5 ng/mL IFN-γ (A) or 100 ng/mL LPS +5 ng/mL IFN-γ (B). Results are presented as % of MAP2 immunostaining vs each control. Bars are means + SEM of three to five independent experiments. *p<0.05 and ***p<0.001 vs each control, unpaired Student’s *t*-test. (C-H) MAP-2 immunostaining in control neuron-BV2 co-cultures (C, D) and co-cultures treated with LPS 100 ng/mL + IFN-γ 0.5 ng/mL for 24 h (E) or 48 h (G) and with LPS 100 ng/mL + IFN-γ 5 ng/mL for 24 h (F) or 48 h (H). Bar = 100 µm.

### Cell Cultures

#### BV2 cells

The mouse microglial cell line BV2 (generated from primary mouse microglia transfected with a v-raf/v-myc oncogene, [17]) was cultured in RPMI-1640 medium (Invitrogen, Carlsbad, CA), supplemented with 0.1% penicillin-streptomycin (Invitrogen) and 10% heat-inactivated foetal bovine serum (FBS, Invitrogen). Cells were maintained at 37°C in a 5% CO_2_ humidified atmosphere. For nitrite assay and tumor necrosis factor (TNF)-α production, cells were seeded at a density of 5×10^4^ cells/mL (1.6×10^4^ cells/cm^2^), and for protein extraction at 10^5^ cells/mL (2.4×10^6^ cells/cm^2^).

**Figure 2:**
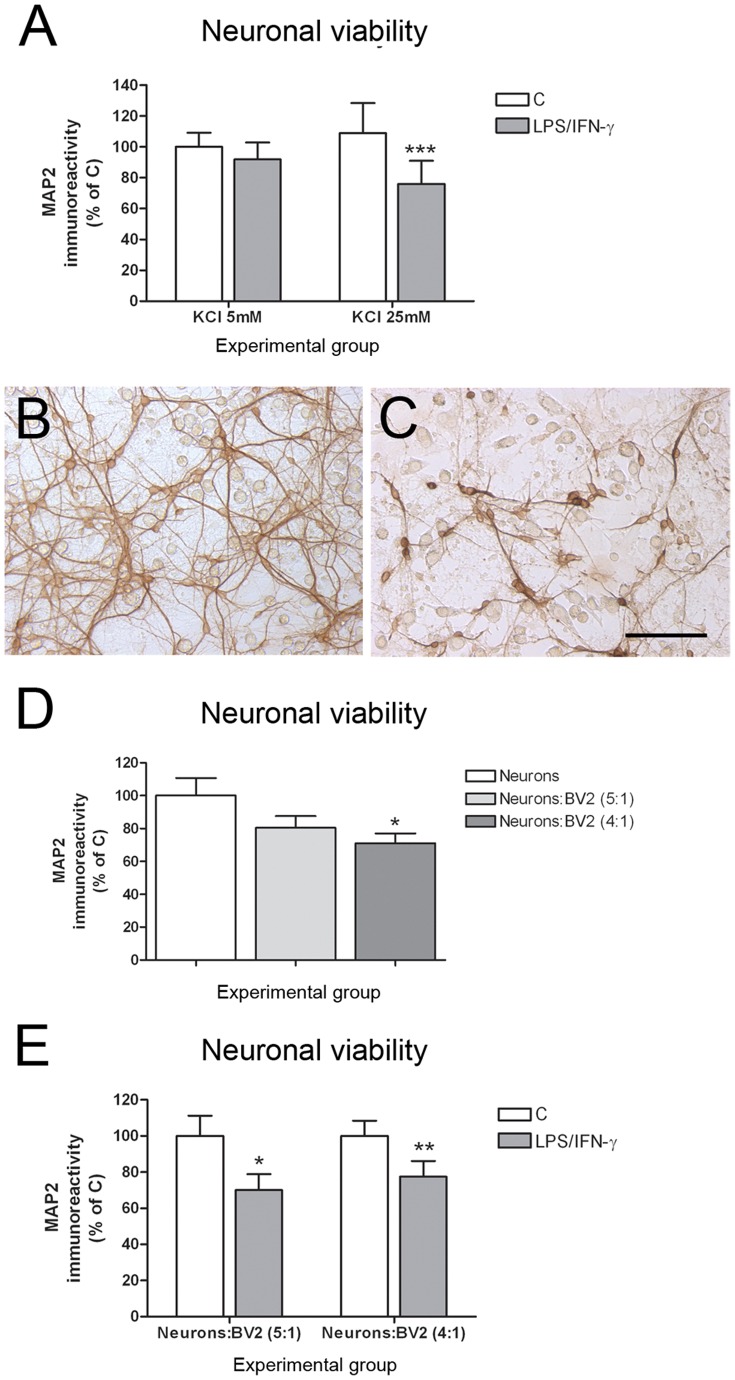
Increased extracellular K^+^ concentration enhanced reactive glia-induced. neurotoxicity in neuron-BV2 co-cultures. (A) Neuronal viability (MAP2-ABTS-ELISA assay) in neuron-BV2 co-cultures grown in 5 mM and 25 mM KCl 24 h after 100 ng/mL LPS +0.5 ng/mL IFN-γ treatment. Results are presented as % of MAP2 immunostaining in control co-cultures grown in 5 mM KCl. Bars are means + SEM of four independent experiments. ***p<0.001 vs respective control; two-way ANOVA (repeated measures) and Bonferroni post-test. (B) MAP2 immunostaining in control (B) and 100 ng/mL LPS +0.5 ng/mL IFN-γ treated co-cultures grown in high K^+^. Bar = 100 µm. (D) Increasing BV2:neuron ratio results in neurotoxicity in control neuron-BV2 co-cultures. (E) Increasing BV2:neuron ratio does not result in increased neurotoxicity in 100 ng/mL LPS +0.5 ng/mL IFN-γ treated co-cultures.

#### Primary cultures

Primary microglia-enriched cultures were obtained from primary mixed glial cultures from 2- to 4-day-old C57BL/6 mice. To obtain mixed glial cultures, cerebral cortices were dissected, carefully stripped of their meninges, and digested with 0.25% trypsin-EDTA solution (Invitrogen) for 25 min at 37°C. Trypsinization was stopped by adding an equal volume of culture medium, to which 0.02% deoxyribonuclease I (Sigma-Aldrich, St. Louis, MO) was added. The culture medium consisted of Dulbecco’s modified Eagle medium-F-12 nutrient mixture (Invitrogen) supplemented with 10% FBS, 0.1% penicillin-streptomycin (Invitrogen), and 0.5 µg/mL amphotericin B (Fungizone®, Invitrogen). Cells were pelleted (5 min, 200 g), resuspended in culture medium, and brought to a single cell suspension by repeated pipetting followed by passage through a 100 µm-pore mesh. Cells were seeded at a density of 3.5×10^5^ cells/mL (1.2×10^5^ cells/cm^2^) and cultured at 37°C in a 5% CO_2_ humidified atmosphere. Medium was replaced every 5–7 days. Microglial cultures were prepared by the mild trypsinization method previously described in our group [18]. Briefly, after 19–21 days *in vitro* (DIV) mixed glial cultures were treated for 30 min with 0.06% trypsin in the presence of 0.25 mM EDTA and 0.5 mM Ca^2+^. This resulted in the detachment of an intact layer of cells containing virtually all the astrocytes, leaving a population of firmly attached cells identified as >98% microglia. The microglial cultures were treated 24 h after isolation by this procedure.

**Figure 3:**
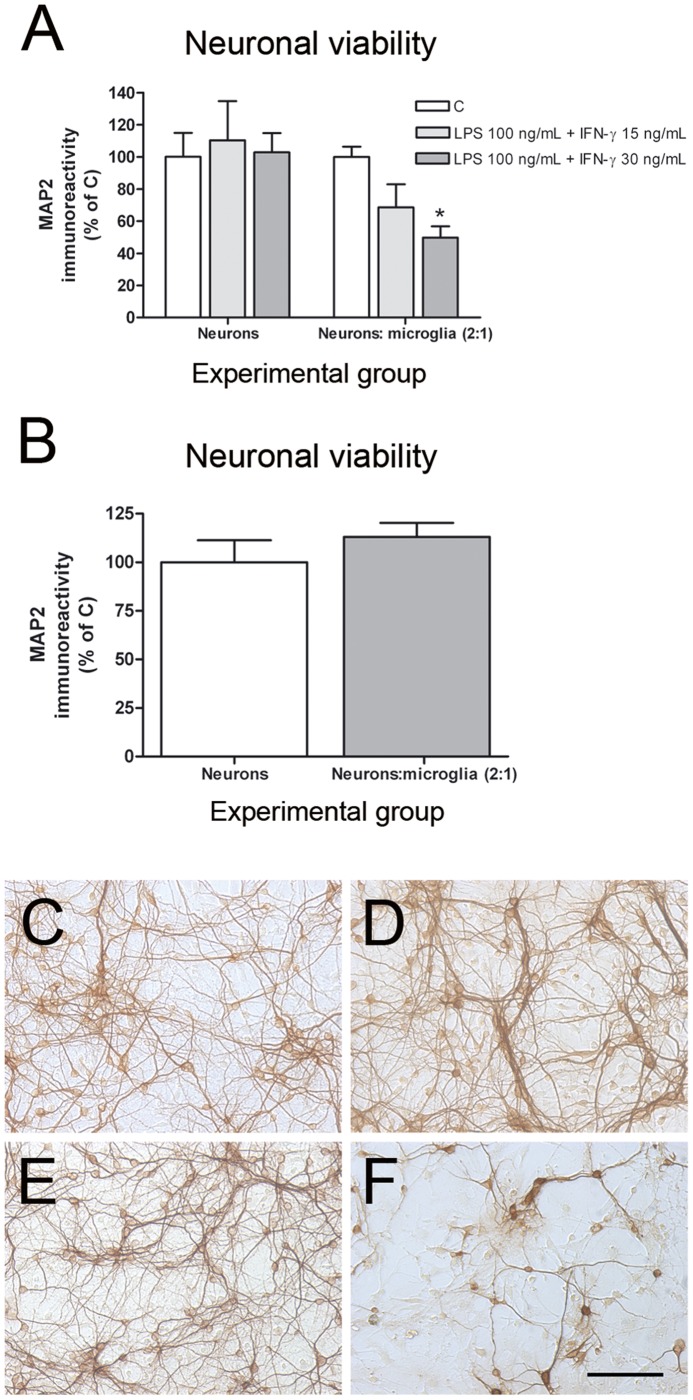
Glial activation and resulting neurotoxicity in neuron-primary microglia co-cultures. (A) Neuronal viability (MAP2-ABTS-ELISA assay) in neuronal cultures and neuron-primary microglia co-cultures 48 h after treatment with 100 ng/mL LPS and increasing concentrations of IFN-γ (15 and 30 ng/mL). (B) The addition of microglial cells at a microglia:neuron ratio of 1∶2 did not result in neurotoxicity after 48 h. Results are presented as % of MAP2 immunostaining vs each control. Bars are means + SEM of 3–4 independent experiments. *p<0.05 vs control; one-way ANOVA and Newman-Keuls post-test. MAP-2 immunocytochemistry in neuronal cultures (C-D) and neuron-primary microglia co-cultures (E-F) in control conditions (C and E) and 48 h after 100 ng/mL LPS +30 ng/mL IFN-γ (D and F).

Primary cortical neuronal cultures were prepared from C57BL/6 mice on embryonic day 16 according to the method described by Frandsen and Schousboe [19]. Cells were seeded at a density of 8×10^6^ cells/mL (2.6×10^5^ cells/cm^2^) in poly-D-lysine hydrobromide (Sigma-Aldrich) coated 48-well culture plates and cultured at 37°C in a 5% CO_2_ humidified atmosphere in Dulbecco’s modified Eagle medium, (Biochrom AG, Berlin, Germany) with 10% FBS, 26.2 mM NaHCO_3_ and 31 mM glucose, 1 µg/mL insulin (Sigma-Aldrich,), 30 mg/mL penicillin G (Sigma-Aldrich), 1 mg/mL aminobenzoic acid and 0.2 mM L-glutamine (Sigma-Aldrich). In some experiments KCl was added to the culture media to reach a potassium concentration of 25 mM.

**Figure 4:**
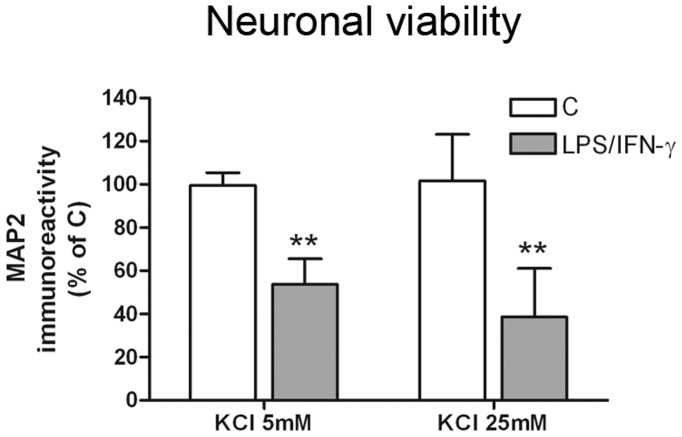
High extracellular K^+^ concentration did not potentiate reactive glia-induced neurotoxicity in neuron-primary microglia co-cultures. Neuronal viability (MAP2-ABTS-ELISA assay) in neuron-primary microglia co-cultures grown in 5 mM and 25 mM KCl and treated with 100 ng/mL LPS +30 ng/mL IFN-γ for 24 h. Results are presented as % of MAP2 immunostaining in control co-cultures grown in 5 mM KCl. Bars are means + SEM of four independent experiments. **p<0.01 vs each control; two-way ANOVA (repeated measures) and Bonferroni post-test.

These cultures contained 76% neurons (positive for microtubule-associated protein 2, MAP2, immunostaining), 17% astrocytes (positive for glial fibrillary acidic protein immunostaining), <1% microglial cells (positive for CD11b immunostaining), and 6% other cell types. These neuron-glia cultures are called “neuronal cultures” here. Neuronal cultures were used at 5 DIV.

**Figure 5:**
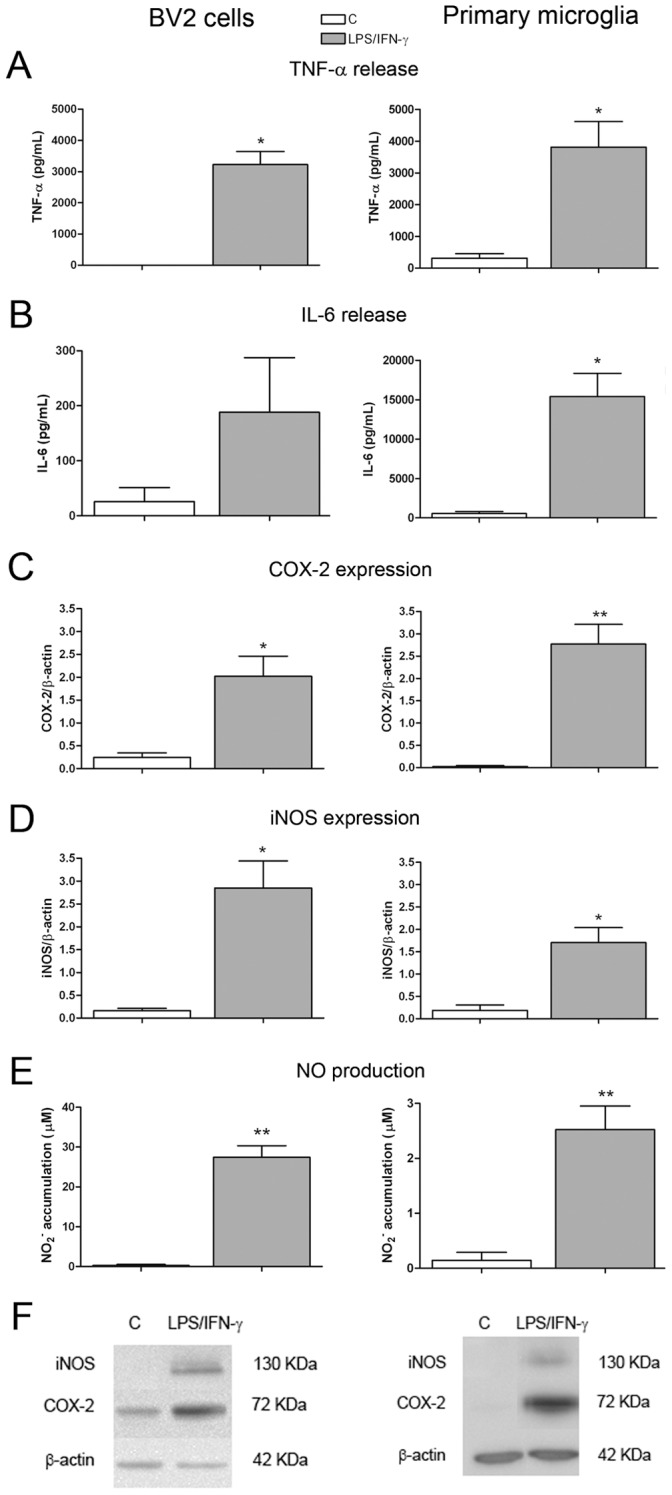
Pro-inflammatory response in BV2 and primary microglia cell cultures in response to LPS/IFN-γ. BV2 cells were treated 100 ng/mL of LPS +0.5 ng/mL of IFN-γ and primary microglial cells with 100 ng/mL LPS +30 ng/mL IFN-γ, and different pro-inflammatory parameters were measured. TNF-α (A) and IL-6 (B) levels were determined in the culture medium 24 h after treatment. COX-2 (C) and iNOS protein expression (D) were measured by western blot 12 h after treatment, and data normalized by β-actin. NO production (E) was determined in the culture medium 24 h after treatment. Bars are means + SEM of four to six independent experiments. *p<0.05 and **p<0.01 vs control; paired Student’s *t* test. (F) shows representative western blots.

#### Co-cultures

For neuron-BV2 co-cultures BV2 cells growing in T75–T150 culture flasks were gently scraped in neuronal culture medium, and aliquots of the cell suspension (50 µl/well) were seeded on top of 5 DIV primary neuronal cells at different final densities (1.5×10^5^ cells/mL or 5×10^4^ cells/cm^2^, and 2×10^5^ cells/mL or 6.7×10^4^ cells/cm^2^, referred in the text as 1∶5 and 1∶4 BV2:neuron ratios respectively).

**Figure 6:**
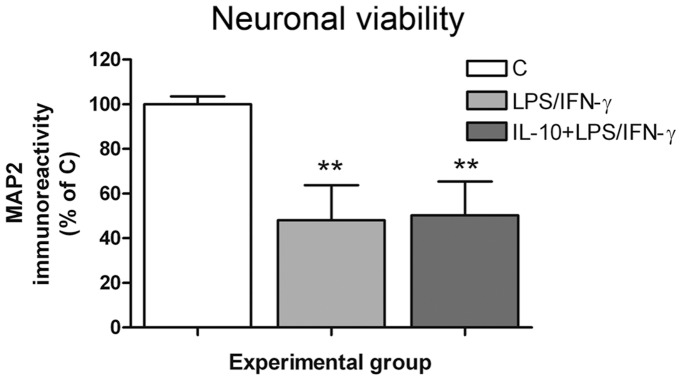
IL-10 pre-treatment did not inhibit the neurotoxicity induced by reactive microglia in neuron-primary microglia co-cultures. Neuron-primary microglia co-cultures were treated with 100 ng/mL LPS +30 ng/mL IFN-γ for 48 h in the presence or absence of 50 ng/mL of IL-10 administered 1 h prior to LPS/IFN-γ. Evaluation of neuronal viability by MAP2-ABTS-ELISA assay. Results are presented as % of MAP2 immunostaining in control co-cultures. Bars are means + SEM of four independent experiments. **p<0.01 vs control; one-way ANOVA (repeated measures) and Newman-Keuls post-test.

**Figure 7:**
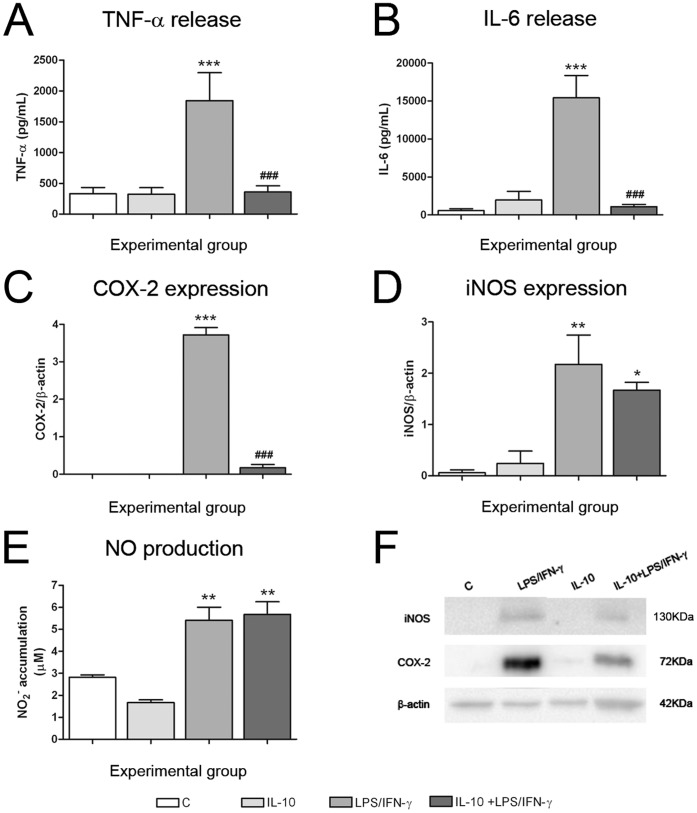
IL-10 pre-treatment inhibited the pro-inflammatory response induced by LPS/IFN-γ in primary microglia cultures. Primary microglial cultures were treated with 100 ng/mL LPS +30 ng/mL IFN-γ for 24 h in the presence or absence of 50 ng/mL IL-10 administered 1 h prior to LPS/IFN-γ. TNF-α (A) and IL-6 (B) levels were determined in the culture medium 24 h after treatment. (C) COX-2 and (D) iNOS protein expression were measured by western blot 24 h after treatment, and data normalized by β-actin. (E) NO production was determined in the culture medium 24 h after treatment. Bars are means + SEM of three to four independent experiments. *p<0.05, **p<0.01 and ***p<0.001 vs control; ###p<0.001 vs LPS/IFN-γ; one-way ANOVA (repeated measures) and Newman-Keuls post-test. (F) shows a representative western blot.

For neuron-primary microglia co-cultures microglial cultures were obtained as described above. After the isolation, microglia-enriched cultures were incubated with 0.25% trypsin for 10 min at 37°C. Trypsinization was stopped by adding the same volume of culture medium with 10% FBS. Cells were gently scraped and centrifuged for 5 min at 200 g. Pellet was resuspended in neuronal culture medium and aliquots of the cell suspension (50 µl/well) were seeded on top of 5 DIV primary neuronal culture at different final densities (4×10^5^ cells/mL or 13×10^4^ cells/cm^2^, 2×10^5^ cells/mL or 6.7×10^4^ cells/cm^2^, and 1.5×10^5^ cells/mL or 5×10^4^ cells/cm^2^, referred in the text as 1∶5, 1∶4 and 1∶2 microglia:neuron ratios respectively).

**Figure 8:**
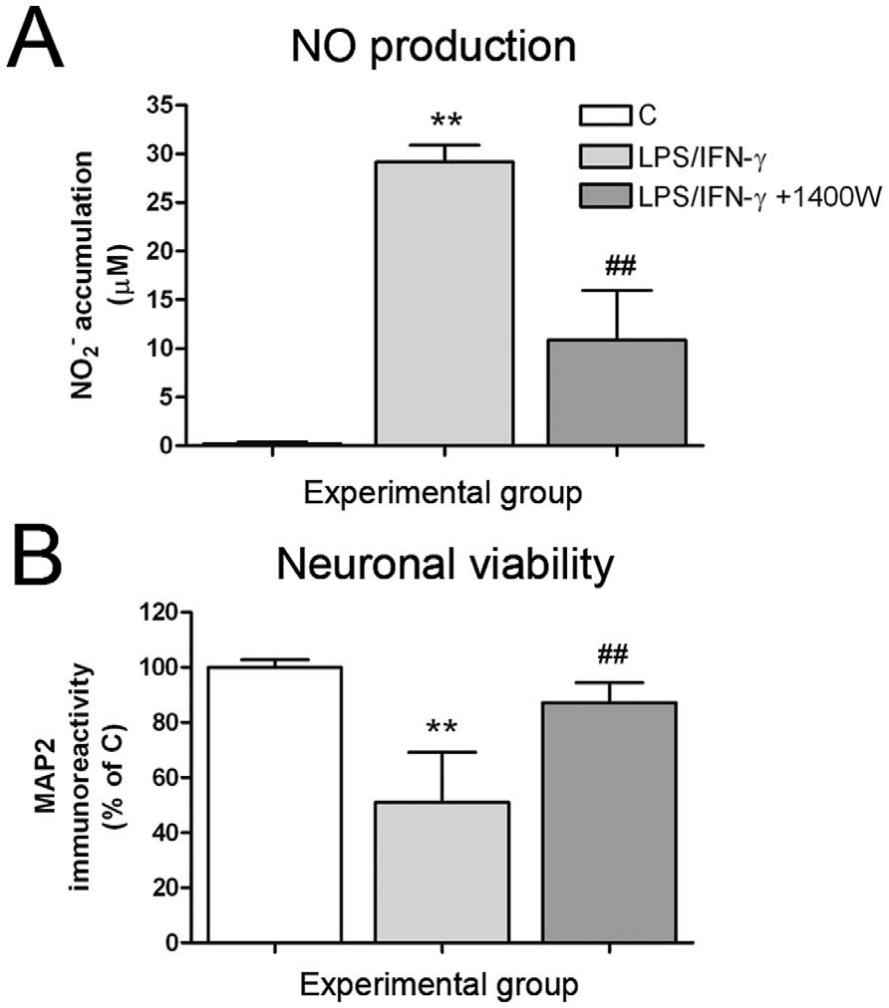
The iNOS inhibitor 1400 W prevented the neurotoxicity induced by reactive microglia in neuron-primary microglia co-cultures. Neuron-primary microglia co-cultures were treated with 100 ng/mL LPS +30 ng/mL IFN-γ for 48 h in the presence or absence of 1400 W (10 µM) co-administered with LPS/IFN-γ. (A) NO production 48 h after LPS/IFN-γ treatment. (B) Evaluation of neuronal viability by MAP2-ABTS-ELISA assay. Results are presented as % of MAP2 immunostaining in control co-cultures. Bars are means + SEM of three independent experiments. **p<0.01 vs control; ##p<0.01 vs LPS/IFN-γ; one-way ANOVA (repeated measures) and Newman-Keuls post-test.

### Treatments

#### BV2 cells

One day after seeding, the culture medium was replaced by fresh RPMI medium. One day later, cells were treated with 100 ng/mL LPS (Sigma-Aldrich, *E. coli* serotype 026:B6) and 0.5 ng/mL recombinant mouse IFN-γ (Sigma-Aldrich).

#### Mouse primary microglia

One day after isolation, microglia-enriched cultures were treated with 100 ng/mL LPS and 30 ng/mL IFN-γ.

A stock solution of 50 µg/mL IL-10 (Peprotech, Rocky Hill, NJ) was prepared in DMEM:F-12 culture media and stored at −20°C. It was added to the culture media 1 h prior to LPS/IFN-γ treatment, at a final concentration of 50 ng/mL.

#### Neuron-BV2 co-cultures

100 ng/mL LPS and 0.5 or 5 ng/mL IFN-γ were added to the culture medium 2 h after seeding BV2 cells on top of neuronal cultures.

#### Neuron-primary microglia co-cultures

100 ng/mL LPS and 15 or 30 ng/mL IFN-γ were added to the culture medium one day after seeding primary microglial cells on top of neuronal cultures.

1400 W dihydrochloride (Tocris, Ellisville, MO) was prepared as a 900 µM stock solution in milliQ water and stored at −20°C. It was added to the culture media at the same time as LPS/IFN-γ treatment, at a final concentration of 10 µM.

### Nitrite Assay

NO production was assessed by the Griess reaction. Briefly, culture supernatants were collected 24 h after LPS/IFN-γ treatment, and incubated with equal volumes of Griess reagent for 10 min at room temperature. Optical density at 540 nm was measured using a microplate reader (Multiskan spectrum, Thermo electron corporation, Waltham, CA). Nitrite concentration was calculated from a sodium nitrite standard curve.

### Tumor Necrosis Factor (TNF)-α Assay

The amount of TNF-α released into the culture medium was measured using an ELISA kit specific for mouse TNF-α (Mouse TNFα ELISA Ready-SET-Go kit, eBioscience, San Diego, CA), following manufacturer’s instructions. Culture supernatants were collected 24 h after LPS/IFN-γ treatment and stored at −80°C until assayed for TNF-α content.

### IL-6 Assay

The amount of IL-6 released into the culture medium was measured using an ELISA kit specific for mouse IL-6 (Murine IL-6 Eli-pair, Diaclone, Besançon, France), following the manufacturer’s instructions. Culture supernatants were collected 24 h after LPS/IFN-γ treatment and stored at −80°C until assayed for IL-6 content.

### Isolation of Total Proteins

Total protein extracts were obtained as previously described [20]. Inducible nitric oxide synthase and cyclooxygenase-2 expression were measured in total protein extracts from BV2 and primary microglial cells 12 h or 24 h after treatments, using 2 or 3 wells from 6-well plates for BV2 cells or mouse primary microglia respectively for each experimental condition. Protein amount was measured by Lowry assay (Total Protein kit micro-Lowry, Sigma-Aldrich).

### Western Blot Analysis

Around 20–40 µg of protein, of denatured (100°C for 5 min) protein total extracts, was subjected to SDS-PAGE on a 7% polyacrylamide gel, together with a molecular weight marker (Fullrange Rainbow Molecular Weight Marker, Amersham, Buckinghamshire, UK), and transferred to a PVDF membrane (Millipore, Bedford, MA). After washing in Tris-buffered saline (TBS: 20 mM Tris, 0.15 M NaCl, pH 7.5) for 5 min, dipping in methanol for 10 s and air drying, the membranes were incubated with primary antibodies overnight at 4°C: polyclonal rabbit anti-COX-2 (1∶2000, Santa Cruz Biotechnology), polyclonal rabbit anti-NOSII (1∶200, Chemicon, Temecula, CA) and monoclonal mouse anti-ß actin (1∶40000, Sigma-Aldrich) diluted in immunoblot buffer (TBS containing 0.05% Tween-20 and 5% non-fat dry milk). Then, the membranes were washed twice in 0.05% Tween-20 in TBS for 15 s and incubated in horseradish peroxidase (HRP)-labelled secondary antibodies for 1 h at room temperature: donkey anti-rabbit (1∶5000, Amersham, Buckinghamshire, UK) and goat anti-mouse (1∶5000, Santa Cruz Biotechnology). After extensive washes in 0.05% Tween-20 in TBS, they were incubated in ECL-Plus (Amersham) for 5 min. Membranes were then exposed to the camera of a VersaDoc System (Bio-Rad Laboratories, Hercules, CA), and pixel intensities of the immunoreactive bands were quantified using the % adjusted volume feature of Quantity One 5.4.1 software (Bio-Rad Laboratories). Data are expressed as the ratio between the intensity of the protein of interest band and the loading control protein band (β-actin).

### Immunocytochemistry

Cultured cells were fixed with 4% paraformaldehyde in PBS for 20 min at room temperature. Cells were permeated and endogenous peroxidase activity was blocked by incubation with 0.3% H_2_O_2_ in methanol for 10 min. Non-specific staining was blocked by incubating the cells with 10% normal goat serum in PBS containing 1% BSA for 20 min at room temperature. The cells were then incubated with monoclonal mouse anti-MAP2 primary antibody (1∶4000, Sigma-Aldrich) overnight at 4°C, and with biotinylated horse anti-mouse secondary antibody (1∶200, Vector, Burlingame, CA, USA) for 1 h at room temperature. Following incubation with ExtrAvidin-HRP (1∶500, Sigma-Aldrich) for 1 h at room temperature, colour was developed with diaminobenzidine (Sigma-Aldrich). The antibodies were diluted in PBS containing 1% BSA and 10% normal horse serum (Vector).

Microscopy images were obtained with an Olympus IX70 microscope (Olympus, Okoya, Japan) and a digital camera (CC-12, Cell^∧^P Olympus Soft Imaging System GmbH, Hamburg, Germany).

### Assessment of Neuronal Viability

Neuronal viability was evaluated by MAP2 immunostaining using ABTS (2,3′-azino-bisethylbenzothiazoline-6-sulphonic acid) as previously described [21]. Briefly, MAP2 staining was performed using peroxidase labelling as described above, but colour was developed using the ABTS Peroxidase Substrate Kit (Vector) following the manufacturer’s instructions. Three wells per experimental condition were processed, and each experimental condition was repeated at least three times. One of the wells was processed without primary antibody and used as background control: its average absorbance was subtracted from sample absorbance to calculate neuronal viability. Neuronal viability was expressed as a percentage of control levels.

### Data Presentation and Statistical Analysis

Results are presented as the means + SEM. Student’s two-tailed *t*-test was used when two groups were considered. One-way ANOVA followed by Newman-Keuls post-test or two-way ANOVA followed by Bonferroni post-test were performed when three or more experimental groups were compared. Values of p<0.05 were considered statistically significant.

## Results

### Activation of Microglia with LPS/IFN-γ had a Neurotoxic Effect: Neuron-BV2 Co-cultures as an Experimental Model of Neuroinflammation

As an experimental model of neuroinflammation *in vitro*, we cultured BV2 cells on top of primary cortical neurons (at a ratio of 1∶5) and we treated the co-cultures with 100 ng/mL LPS and increasing concentrations of IFN-γ (0.5 and 5 ng/mL). The induction of neurotoxicity in response to glial activation was evaluated 24 or 48 h after treatment. LPS alone or IFN-γ alone had no effect on neuronal viability in the co-cultures (Fig. S1). However, although LPS +0.5 ng/mL IFN-γ treatment did not affect neuronal viability at 24 h, significant neurotoxicity was observed at 48 h (Fig. 1A), measured as % of MAP2 immunostaining versus control as explained in Material and Methods. An increased concentration of IFN-γ (5 ng/mL) markedly enhanced neurotoxicity in the co-cultures at 48 h (83±17% neuronal death using 5 ng/mL versus 44±11% neuronal death using 0.5 ng/mL), in the absence of any effect on neuronal viability at 24 h (Fig. 1B). Figure 1C–H shows MAP2 immunocytochemistry in neuron-BV2 co-cultures treated with LPS +0.5 ng/mL IFN-γ (left column) or LPS +5 ng/mL IFN-γ (right column). A clear decrease in immunolabelling in the neuronal network was observed 48 h after both treatments (Fig. 1G–H), but not at 24 h (Fig. 1E–F). LPS + IFN-γ treatments did not induce neurotoxicity in neuronal cultures (data not shown).

### The Neurotoxic Potential of Reactive BV2 Cells Increased in the Presence of High Extracellular K^+^ Concentration

Acute brain injury is associated with increased extracellular K^+^ concentration, which has been reported to enhance neurotoxicity induced by activated glial cells *in vitro* [22], suggesting that extracellular K^+^ modulates neuroinflammation and the resulting neurotoxicity. We determined whether an increase in K^+^ concentration in the culture media could potentiate the induction of neuronal death in our experimental model of neuroinflammation *in vitro*. BV2 cells were added to neuronal cultures grown in the presence of 5 mM KCl (low K^+^) or 25 mM KCl (high K^+^). The effect of 100 ng/mL LPS +0.5 ng/mL IFN-γ on neuronal viability was determined 24 h after treatment, when neuronal death was not observed in co-cultures grown in low K^+^ (Fig. 1). No differences in neuronal viability were observed between control co-cultures grown in high K^+^ and low K^+^ (Fig. 2A). However, significant neuronal death was observed 24 h after LPS/IFN-γ treatment only in co-cultures grown in high K^+^ (Fig. 2A–C).

We also determined whether an increase in BV2:neuron ratio in the co-cultures could further enhance the neurotoxicity of 100 ng/mL LPS +0.5 ng/mL IFN-γ treatment. However, increasing the BV2:neuron ratio from 1∶5 to 1∶4 induced significant neurotoxicity in the control co-cultures (Fig. 2D), without an increase in neurotoxicity in the LPS/IFN-γ treated co-cultures (Fig. 2E).

### Comparing Neuron-BV2 Co-cultures with Neuron-primary Microglia Co-cultures to Establish an Experimental Model of Neuroinflammation

The use of cell lines has many advantages, especially in the case of microglial cells, as regards the amount of cells that can be easily and rapidly obtained. However, in some cases cell lines respond to certain stimuli in a different way than primary cultures. Consequently, we studied possible differences between neuron-BV2 and neuron-primary microglia co-cultures in our model of neuroinflammation. Primary microglial cells were seeded on the top of neuronal cultures at different microglia:neuron ratios, and neuronal death was assessed after inducing glial activation with 100 ng/mL LPS and increasing concentrations of IFN-γ (0.5 and 5 ng/mL). Unlike neuron-BV2 co-cultures, no significant neurotoxicity was observed in neuron-microglia co-cultures 48 h after treatment using primary microglia:neuron ratios of 1∶5, 1∶4, nor at a primary microglia:neuron ratio of 1∶2 (data not shown). When IFN-γ concentration was increased to 15 and 30 ng/mL, significant neurotoxicity was observed in neuron-primary microglia co-cultures at a primary microglia:neuron ratio of 1∶2 (Fig. 3A). No neurotoxic effect of the treatments was observed in neuronal cultures (Fig. 3A). No effect in neuronal viability was observed following the addition of microglial cells in control co-cultures (Fig. 3B). MAP2 immunocytochemistry shows no alterations in neuronal cultures (Fig. 3C–D) and a clear decrease of staining in the neuronal network in neuron-primary microglial co-cultures 48 h after 100 ng/mL LPS +30 ng/mL IFN-γ-treatment (Fig. 3E–F).

We also examined whether an increase in KCl concentration in the culture media could potentiate the induction of neuronal death in our experimental model of neuroinflammation *in vitro* using primary microglial cells. Primary microglial cells were added to neuronal cultures grown in the presence of 5 mM KCl (low K^+^) or 25 mM KCl (high K^+^), and the effect of 100 ng/mL LPS +30 ng/mL IFN-γ on neuronal viability was determined 48 h after treatment. Unlike neuron-BV2 co-cultures, no significant differences were observed between neuron-primary microglia co-cultures grown in low K^+^ and high K^+^ (Fig. 4).

### Production of Pro-inflammatory Mediators by LPS/IFN-γ-treated Microglial Cells

In order to identify factors potentially responsible for the neurotoxic effect of activated microglia in the co-cultures in our two models of neuroinflammation, we first characterized the production of pro-inflammatory molecules in BV2 and primary microglial cultures in response to LPS/IFN-γ. We detected an increase in the release of the pro-inflammatory cytokines TNF-α(Fig. 5A) and IL-6 (Fig. 5B) 24 h after treatment both in BV2 and primary microglial cultures. We also observed an increase in the expression of the pro-inflammatory enzymes COX-2 (Fig. 5C and F) and iNOS (Fig. 5D and F) 12 h after treatment in both types of cultures, as well as an increase in NO production (Fig. 5E).

### Pre-treatment with the Anti-inflammatory Cytokine IL-10 did not Reverse LPS/IFN-γ-Induced Neurotoxicity in Neuron-microglia Co-cultures

Once the experimental model of neuroinflammation was established, we tested whether the inhibition of the pro-inflammatory response resulted in neuroprotection, using the anti-inflammatory cytokine IL-10. Nevertheless, IL-10 pre-treatment (50 ng/ml) did not modify the neurotoxic effect of LPS/IFN-γ activated microglia, neither using primary microglia (Fig. 6) nor BV2 cells (data not shown). We then evaluated whether IL-10 pre-treatment was inhibiting the pro-inflammatory response induced by LPS/IFN-γ in microglial cells. Figure 7 shows the results obtained using primary microglia cultures, but similar results were obtained using BV2 cells (data not shown). TNF-α and IL-6 release induced by LPS/IFN-γ were completely inhibited in IL-10 pre-treated cultures (Fig. 7A and B). In addition, drastic inhibition of COX-2 expression was observed in the presence of IL-10 pre-treatment (Fig. 7C and F). However, IL-10 pre-treatment did not modify iNOS expression (Fig. 7D and F) or NO production (Fig. 7E) induced by LPS/IFN-γ.

### NO is the Main Mediator of LPS/IFN-γ-induced Neurotoxicity in Neuron-primary Microglia Co-cultures

Among the different pro-inflammatory parameters that we evaluated in microglial cultures in response to LPS/IFN-γ, NO production and iNOS expression were the only factors not inhibited after IL-10 pre-treatment. Consequently, we next analysed whether the selective inhibition of NO production could reverse LPS/IFN-γ-induced neurotoxicity in our co-cultures, using the specific iNOS inhibitor 1400 W. We confirmed that 1400 W treatment (10 µM) inhibited NO production induced by LPS/IFN-γ in neuron-primary microglia co-cultures (Fig. 8A), and we observed that this effect was associated with the inhibition of LPS/IFN-γ-induced neurotoxicity (Fig. 8B).

## Discussion

In the present work, we have established two experimental models of neuroinflammation *in vitro*, co-culturing primary cortical neurons either with the microglial cell line BV2 or with primary microglia, and treating the co-cultures with LPS/IFN-γ. We have characterized differences in the experimental conditions required in each co-culture to induce neurotoxicity, and subsequently checked the potential neuroprotective effect of the anti-inflammatory cytokine IL-10. Using IL-10, we show that the anti-inflammatory properties of a compound do not always result in neuroprotection. We also show that NO production by reactive glial cells plays a critical role in the neurotoxicity observed in the LPS/IFN-γ-treated co-cultures.

Neuron-glia co-cultures can be used to test anti-inflammatory and neuroprotective properties of selected candidate compounds. Cell lines, either neuronal or glial, or primary cultures can be used for this purpose. The presence of microglial cells in the co-cultures is critical for the neurotoxic effect of glial activation. In fact, we did not detect neurotoxicity in LPS/IFN-γ-treated neuronal cultures when microglial cells were not added, in spite of the significant amount of astrocytes present. However, an indirect contribution of astrocytes in the neurotoxicity observed in the co-cultures cannot be ruled out. As regards the amount of microglial cells present in the co-cultures, we observed that differences in the microglia:neuron ratio have to be taken into account when co-culturing primary neuronal cultures with the microglial cell line BV2 or with primary microglia.We observed that control neuronal cultures survived better with added primary microglial cells than with added BV2 cells. Thus, neuronal death was detected when increasing BV2:neuron ratio from 1∶5 to 1∶4 in untreated co-cultures, while a primary microglia:neuron ratio of 1∶2 did not modify neuronal viability *per se*. The toxic effect of high amounts of BV2 cells on neuronal viability may be due, at least in part, to the high metabolic rate associated with the elevated proliferation rate of the immortalized cell line.

The stimulus used is also critical for the neurotoxic effect of glial activation in the co-cultures. While LPS or IFN-γ alone did not produce neurotoxicity, a neurotoxic effect was observed when the two stimuli were used together to induce glial activation, once a critical concentration of IFN-γ was considered. These results suggest that a critical threshold of glial activation has to be reached to result in a neurotoxic effect. However, different patterns of treatment have to be considered to attain this degree of activation in BV2 and primary microglia. The amount of IFN-γ added to 100 ng/mL LPS necessary to activate the microglial cells in order to induce significant neurotoxicity was higher in neuron-primary microglia co-cultures (15 or 30 ng/mL), where a higher microglia:neuron ratio (1∶2) was also necessary, than in neuron-BV2 co-cultures (0.5 ng/mL IFN-γ). In addition, high extracellular K^+^ levels made neurons more sensitive to neurotoxicity induced by reactive BV2 cells but not to that induced by reactive primary microglia. Differences between reactive BV2 and primary microglia are also manifested when looking at the production of pro-inflammatory factors in response to LPS/IFN-γ treatment. Although the results obtained can not be quantitatively compared due to the differences in cell density between BV2 and primary microglia cultures, both types of culture produce comparable amounts of TNF-α, but NO production is higher and IL-6 lower in BV2 cultures than in primary microglia cell cultures. All these pro-inflammatory molecules are potentially neurotoxic by themselves or in combination, but the final neurotoxicity observed in the co-cultures may result both from the contribution of microglia secreted products and the interaction between the different cell types present, which in turn will depend on the microglia cell type (BV2 or primary microglial cells).

Both neuron-BV2 and neuron-primary microglia co-cultures can be useful to establish an *in vitro* model of neuroinflammation, and both of them have particular advantages. On the one hand, apart from constituting an alternative to reduce the use of experimental animals [23], the use of the BV2 cell line makes large amounts of microglial cells available when necessary, which overcomes the special difficulty of obtaining significant amounts of primary microglial cells. On the other hand, the use of primary microglial cells is closer to the physiological situation. Nevertheless, since immortalization significantly affects a cell’s biology, and differences in BV2 and primary microglia responses to different stimuli have been described [24]–[25], the use of primary cultures to validate results obtained using BV2 is recommended.

In glial cultures, different pro- and anti-inflammatory cytokines, as well as reactive oxygen and nitrogen species, are produced in response to LPS treatment. These factors are involved in the inflammatory response of reactive glial cells and the resolution of the inflammation. IL-10 is a classic immunoregulatory and anti-inflammatory cytokine. Several authors have shown the inhibitory effect of IL-10 treatment on the production of pro-inflammatory cytokines by reactive glia in response to LPS [26]–[28]. In accordance with these results, we observed that IL-10 pre-treatment clearly inhibited IL-6 and TNF-α production as well as COX-2 expression induced by LPS/IFN-γ treatment in microglial cultures (primary microglia and BV2 cells). Contradictory results have been described regarding IL-10 inhibition of NO production in LPS-treated glial cells [26], [27], [29], [30]. IL-10 pre-treatment did not inhibit NO production or iNOS expression in LPS/IFN-γ-treated microglial cultures. These results show a differential regulation of pro-inflammatory molecules by IL-10 in reactive mouse microglia.

Different studies demonstrate the neuroprotective effect of IL-10 *in vivo* or *in vitro* against LPS- [31], [32], glutamate- [33], oxygen-glucose-deprivation- [34] and transection- induced neuronal damage [35]. However, a lack of neuroprotective effect of IL-10 against neuronal damage, conflicting results, or even a deleterious effect of IL-10, have also been reported in different experimental *in vivo* approaches [32], [36]–[38]. The neuroprotective action of Il-10 is attributed, at least in part, to the inhibition of pro-inflammatory microglial activation, although a direct effect of the cytokine on neuronal viability has also been described. Although we observed that IL-10 inhibited the production of TNF-α and IL-6 and the induction of COX-2 in LPS/IFN-γ-treated microglial cells, we did not observe neuroprotection after IL-10 pre-treatment in LPS/IFN-γ-treated mouse neuron-microglia co-cultures. As IL-10 pre-treatment did not inhibit NO production induced by LPS/IFN-γ and the iNOS inhibitor 1400 W had a strong neuroprotective effect in our experimental model, the lack of effect of IL-10 on NO production may be responsible for the absence of neuroprotection. These results suggest that, although a synergistic action of the different pro-inflammatory molecules produced by reactive glial cells cannot be ruled out, NO production plays a major role in reactive microglia induced neurotoxicity. NO can induce neuronal cell damage by disrupting the neuronal mitochondrial electron transport chain [39], [40]. In addition, NO may react with superoxide ion produced by glial NADPH oxidase to generate peroxynitrite [41], which in turn induces neurotoxicity through the inhibition of mitochondrial respiration, the induction of neuronal apoptosis and the induction of glutamate release resulting in excitotoxicity [41], [42]. NO can also cause protein nitration and S-nitrosylation, resulting in loss of function and neurotoxicity [43]. Consequently, NO production by reactive glial cells probably contributes to neurotoxicity either directly or through peroxynitrite and is a potential target to be considered in neuroprotective strategies aimed to reduce neuronal death associated with neuroinflammation.

In conclusion, neuron-BV2 and neuron-primary microglia LPS/IFN-γ-treated co-cultures are a useful tool with which to study the potential neuroprotective effect of anti-inflammatory agents. However, different experimental conditions have to be established when using primary microglia or BV2 cells. In addition, results obtained using BV2 cells have to be confirmed with primary microglia to discard possible modifications in the behaviour of the microglial immortalized cells. We show that the anti-inflammatory properties of a compound do not always result in neuroprotection and that, among the anti-inflammatory agents, special attention should be paid to those compounds that inhibit NO production. Nevertheless, although *in vitro* studies are a useful tool to test anti-inflammatory and neuroprotective properties of compounds, the *in vivo* context in each situation of CNS injury will finally define the anti-inflammatory and neuroprotective effects of a compound.

## Supporting Information

Figure S1 **Absence of neurotoxicity in neuron-BV2 co-cultures after LPS or IFN-γ-treatment.** (A) Neuronal viability (MAP2-ABTS-ELISA assay) in neuron-BV2 co-cultures 24 h or 48 h after treatment with 100 ng/mL LPS, 0.5 ng/mL IFN-γ or 5 ng/mL IFN-γ. Results are presented as % of MAP2 immunostaining vs each control. Bars are means + SEM of three independent experiments. One way ANOVA p>0.05. MAP2 immunostaining in control neuron-BV2 cocultures (B, C) and co-cultures treated with 100 ng/mL LPS (D, E), 0.5 ng/mL IFN-γ (F, G) or 5 ng/mL IFN-γ (H, I) for 24 h (left column) or 48 h (right column). Bar = 100 µm.
(TIF)
