# Supplementary figures and images for: Modelling Neuroinflammation In Vitro: A Tool to Test the Potential Neuroprotective Effect of Anti-Inflammatory Agents
Source: PLoS One. 2012 Sep 20;7(9):e45227. doi: 10.1371/journal.pone.0045227 (PMC3447933; doi:10.1371/journal.pone.0045227)

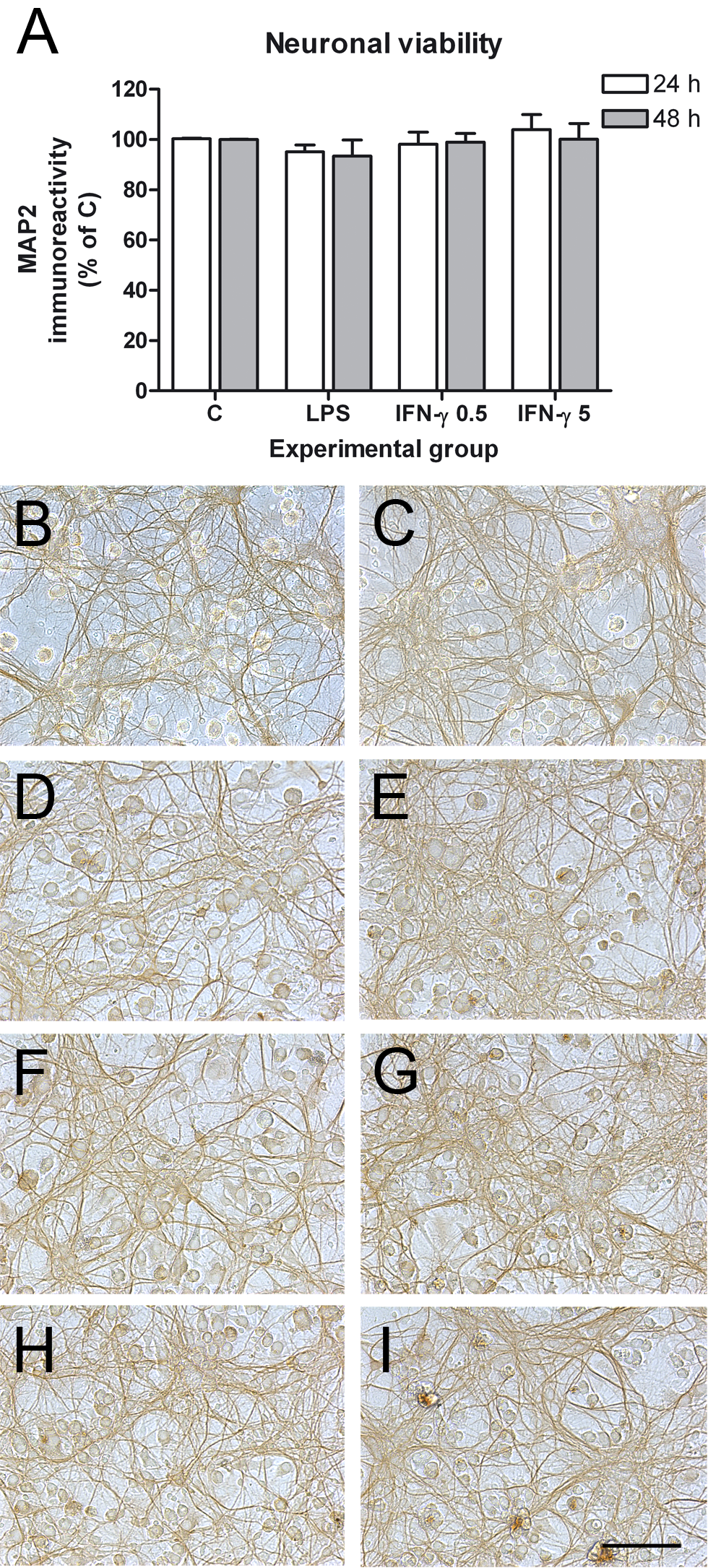

Supplement: Figure S1 — Absence of neurotoxicity in neuron-BV2 co-cultures after LPS or IFN-γ-treatment. (A) Neuronal viability (MAP2-ABTS-ELISA assay) in neuron-BV2 co-cultures 24 h or 48 h after treatment with 100 ng/mL LPS, 0.5 ng/mL IFN-γ or 5 ng/mL IFN-γ. Results are presented as % of MAP2 immunostaining vs each control. Bars are means + SEM of three independent experiments. One way ANOVA p>0.05. MAP2 immunostaining in control neuron-BV2 cocultures (B, C) and co-cultures treated with 100 ng/mL LPS (D, E), 0.5 ng/mL IFN-γ (F, G) or 5 ng/mL IFN-γ (H, I) for 24 h (left column) or 48 h (right column). Bar = 100 µm. (TIF) [file pone.0045227.s001.tif]
